# Supplementary material for: Identifying a Minor Histocompatibility Antigen in Mauritian Cynomolgus Macaques Encoded by APOBEC3C
Source: Front Immunol. 2020 Oct 26;11:586251. doi: 10.3389/fimmu.2020.586251 (PMC7649366; doi:10.3389/fimmu.2020.586251)
Supplement: Supplementary Table 1 — ELISpot responses for 69 T cell clones incubated with 10 (n = 47) or fewer (n = 22) BLC lines. Numbers indicate spot-forming cells (SFCs) per well. Clones that were selected for further analysis are in boxes, with positive responses highlighted in bold. [file Data_Sheet_1.PDF]

Table S1

**T cell clone**	cy0736 blcl	cy0742 blcl	cy0743 blcl	cy0746 blcl	cy0747 blcl	no blcl	cy0870 blcl	cy0872 blcl	cy0874 blcl	cy0878 blcl	cy0739 blcl	no blcl	
cy0737 C1	14	38	5	0	NT	0	NT	NT	NT	NT	NT	NT	
cy0737 C2	410	317	355	249	285	1	6	16	20	69	65	1	
cy0737 C3	2	29	5	3	NT	0	NT	NT	NT	NT	NT	NT	
cy0737 C4	18	58	154	227	83	16	3	2	94	39	117	2	
cy0737 C5	11	63	7	0	NT	0	NT	NT	NT	NT	NT	NT	
cy0737 C6	247	426	254	328	185	10	125	299	375	135	419	13	
cy0737 C7	12	44	17	2	NT	2	**208**	89	**387**	131	140	74	IGHM
cy0737 C8	45	83	198	22	16	20	NT	NT	NT	NT	NT	NT	
cy0737 D2	133	401	310	46	322	31	11	10	22	103	77	0	
cy0737 D3	258	223	38	24	19	22	12	6	24	74	74	0	
cy0737 D4	532	216	12	8	289	4	6	8	11	79	78	0	
cy0737 D5	52	92	228	254	138	4	5	7	14	50	66	0	
cy0737 D6	96	21	31	30	58	37	9	3	8	62	84	0	
cy0737 D7	28	537	20	362	42	13	17	23	30	76	51	11	
cy0737 D8	265	486	360	390	349	1	13	3	69	77	110	0	
cy0737 E1	32	142	221	14	51	8	11	8	17	68	86	0	
cy0737 E2	202	440	386	358	355	3	NT	NT	NT	NT	NT	NT	
cy0737 E3	101	368	136	14	199	3	9	10	23	66	86	0	
cy0737 E4	129	116	TNTC	TNTC	276	171	10	6	24	94	105	1	
cy0737 E5	41	166	100	17	52	4	NT	NT	NT	NT	NT	NT	
cy0737 E6	NT	NT	NT	NT	NT	NT	10	12	21	81	99	0	
cy0737 E7	NT	NT	NT	NT	NT	NT	217	31	56	73	69	19	

	cy0736 blcl	cy0742 blcl	cy0743 blcl	cy0746 blcl	cy0747 blcl	no blcl	cy0870 blcl	cy0872 blcl	cy0874 blcl	cy0878 blcl	cy0739 blcl	no blcl	
cy0738 C1	8	73	7	8	NT	0	17	230	269	48	67	11	
cy0738 C2	20	226	10	233	26	3	14	128	166	58	58	2	
cy0738 C3	234	422	349	367	287	3	17	9	17	101	94	0	
cy0738 C4	119	358	244	241	208	1	12	13	31	94	101	0	
cy0738 C7	442	539	490	568	313	5	16	9	22	77	90	0	
cy0738 C8	158	361	212	162	102	71	NT	NT	NT	NT	NT	NT	
**cy0738 D1**	96	**585**	**514**	**492**	**337**	4	**64**	9	**206**	104	**171**	2	OR4K3
cy0738 D2	28	363	37	565	33	26	11	48	120	44	54	2	
cy0738 D3	510	655	13	579	42	5	8	5	45	64	95	0	
cy0738 D4	235	399	26	211	44	4	18	10	10	75	88	0	
cy0738 D5	32	69	9	11	NT	2	39	35	74	180	97	36	
cy0738 D7	386	680	641	464	429	2	19	9	9	81	116	1	
cy0738 D8	56	162	499	49	84	38	296	13	395	76	99	2	
cy0738 E1	438	166	571	570	501	24	9	10	18	81	81	0	

cy0738 E2	346	423	15	345	35	3	17	11	13	107	99	0
cy0738 E3	206	326	17	173	38	3	9	12	20	77	83	0
cy0738 E4	469	474	431	579	475	2	14	7	29	133	117	0

	cy0736 blcl	cy0742 blcl	cy0743 blcl	cy0746 blcl	cy0747 blcl	no blcl	cy0870 blcl	cy0872 blcl	cy0874 blcl	cy0878 blcl	cy0739 blcl	no blcl	
cy0741 C1	49	51	13	21	NT	23	259	251	323	196	205	158	
cy0741 C2	183	136	3	0	NT	0	191	6	24	81	99	1	
cy0741 C3	8	101	6	41	NT	3	3	10	10	7	6	3	
cy0741 C4	12	213	4	122	NT	1	11	111	110	16	19	7	
cy0741 C5	13	50	18	342	21	18	8	8	7	45	70	0	
cy0741 C6	104	353	37	26	239	22	15	7	22	74	97	0	
cy0741 C7	312	291	3	1	NT	2	11	6	17	39	61	2	
**cy0741 C8**	43	76	**461**	54	75	61	14	4	24	**357**	78	0	Apobec3C
cy0741 D1	375	602	407	428	360	5	9	7	20	63	79	0	
cy0741 D2	165	788	207	833	236	203	143	393	448	156	104	87	
cy0741 D3	55	152	787	33	70	34	354	351	59	110	72	38	
cy0741 D4	161	342	25	184	137	25	17	6	13	71	106	0	
cy0741 D5	402	285	511	400	15	4	6	11	24	95	78	0	
cy0741 D6	597	621	78	35	99	32	NT	NT	NT	NT	NT	NT	
cy0741 D7	28	435	301	150	250	4	11	12	19	76	98	0	
cy0741 D8	NT	NT	NT	NT	NT	NT	13	7	27	98	99	0	

	cy0736 blcl	cy0742 blcl	cy0743 blcl	cy0746 blcl	cy0747 blcl	no blcl	cy0870 blcl	cy0872 blcl	cy0874 blcl	cy0878 blcl	cy0739 blcl	no blcl	
cy0747 C1	186	398	227	180	33	0	8	5	10	57	82	0	
cy0747 C2	36	76	8	23	NT	0	1	4	9	78	66	2	
cy0747 C3	14	226	3	0	NT	0	NT	NT	NT	NT	NT	NT	
cy0747 C4	10	53	8	3	NT	0	17	5	7	41	89	0	
cy0747 C5	7	336	180	301	14	12	11	8	8	62	93	0	
cy0747 C6	14	489	323	359	12	4	163	142	166	68	211	1	
cy0747 C7	326	698	448	374	30	3	43	102	123	115	224	0	
cy0747 C8	774	69	4	891	12	11	8	582	493	567	65	0	
cy0747 D1	106	666	228	666	28	6	0	1	4	10	4	0	
cy0747 D2	88	102	3	1	NT	0	9	0	8	56	76	0	
cy0747 D3	719	848	13	5	36	4	14	4	18	76	88	0	
cy0747 D4	739	853	22	675	30	4	15	10	14	55	83	0	
cy0747 D5	16	**623**	**516**	**606**	47	16	**82**	**172**	**208**	65	**116**	10	IL20RA or MAP3K5
cy0747 D6	NT	NT	NT	NT	NT	NT	132	176	188	63	193	0	

TNTC: Too numerous to count
NT: Not tested
