# Supplementary material for: Identifying a Minor Histocompatibility Antigen in Mauritian Cynomolgus Macaques Encoded by APOBEC3C
Source: Front Immunol. 2020 Oct 26;11:586251. doi: 10.3389/fimmu.2020.586251 (PMC7649366; doi:10.3389/fimmu.2020.586251)
Supplement: Supplementary Table 2 — Segregation analysis of APOBEC3C, IGHM, OR4K3 and IL20RA or MAP3K5. Displayed are the segregation scores for the selected alloreactive T cell clones. MCMs were placed in response or non-response groups based on the recognition of their BLCs by the respective T cell clones. Haplotype indicates the nucleotide SNP present at the chromosome and nucleotide position relative to the Mmu-8.0.1 reference genome. [file Data_Sheet_2.PDF]

Table S2

Top scoring gene: APOBEC3C

Score	Donor	Response group		Non-response group								Chromosome	Nucleotide	Haplotypes		
	cy0741	cy0743	cy0878	cy0736	cy0742	cy0746	cy0747	cy0870	cy0872	cy0874	cy0739			**0**	**1**	**2**
40	1/1	1/2	1/2	1/1	1/1	1/1	1/1	1/1	1/1	1/1	1/1	NC_027902.1	80990325		G	T
40	1/1	1/2	2/2	1/1	1/1	1/1	1/1	1/1	1/1	1/1	1/1	NC_027896.1	33511258		A	C
36	1/1	0/0	0/0	1/1	0/1	1/1	0/1	1/1	1/1	1/1	1/1	NC_027910.1	35901207	C	T	
35	1/1	0/1	0/0	1/1	1/1	1/1	1/1	1/1	1/1	0/1	1/1	NC_027895.1	42138622	G	T	
35	1/1	0/1	0/0	1/1	1/1	1/1	1/1	1/1	1/1	0/1	1/1	NC_027895.1	42142638	C	A	
35	1/1	0/0	0/1	1/1	1/1	1/1	1/1	0/1	1/1	1/1	1/1	NC_027896.1	5131697	C	G	
35	1/1	0/0	0/1	1/1	1/1	1/1	1/1	1/1	1/1	1/1	0/1	NC_027900.1	37161589	A	G	
35	1/1	0/0	0/1	1/1	1/1	1/1	1/1	1/1	1/1	1/1	0/1	NC_027900.1	37161599	G	A	
35	0/1	1/1	0/0	1/1	1/1	1/1	1/1	1/1	1/1	1/1	1/1	NC_027901.1	911116	A	C	
35	0/1	1/1	0/0	1/1	1/1	1/1	1/1	1/1	1/1	1/1	1/1	NC_027901.1	913530	A	G	

IGHM

Score	Donor	Response group		Non-response group								Chromosome	Nucleotide	Haplotypes		
	cy0737	cy0870	cy0874	cy0736	cy0872	cy0746	cy0739	cy0878	cy0742	cy0743				**0**	**1**	**2**
35	1/1	1/2	1/2	1/1	1/1	1/1	1/1	1/1	1/1	1/1		NC_027899.1	168651835	C	T	G
32	1/1	0/1	0/0	1/1	1/1	1/1	1/1	1/1	1/1	1/1		NC_027893.1	134564832	T	C	
31	1/1	1/2	0/2	1/1	0/1	1/1	1/1	1/1	1/1	0/1		NC_027899.1	168887455	T	TGGG	TGAG
31	1/1	0/0	0/0	0/1	1/1	1/1	0/1	1/1	1/1	1/1		NC_027900.1	79964569	C	T	
31	1/1	0/0	0/0	1/1	0/1	0/1	1/1	1/1	1/1	1/1		NC_027908.1	69201503	C	T	
31	0/1	0/0	0/0	1/1	0/1	0/1	1/1	1/1	1/1	1/1		NC_027908.1	69630713	T	C	
31	1/1	0/0	0/0	1/1	0/1	0/1	1/1	1/1	1/1	1/1		NC_027908.1	69734593	G	C	
30	1/1	0/1	0/0	1/1	1/1	1/1	1/1	1/1	0/1	1/1		NC_027893.1	12398244	C	T	
30	2/2	1/2	1/2	1/1	1/1	1/1	1/1	1/2	1/1	1/1		NC_027893.1	21856102		T	C
30	0/1	1/1	0/0	1/1	1/1	1/1	1/1	1/1	1/1	1/1		NC_027893.1	125869464	G	C	

OR4K3

Score	Donor	Response group							Non-response group			Chromosome	Nucleotide	Haplotypes		
	cy0738	cy0742	cy0743	cy0746	cy0747	cy0870	cy0874	cy0739	cy0736	cy0872	cy0878			**0**	**1**	**2**
33	0/0	1/1	1/1	1/1	1/1	1/1	1/1	0/1	0/0	0/0	0/0	NC_027899.1	81192964	G	A	
31	0/0	1/1	1/1	1/1	0/1	1/1	1/1	0/1	0/0	0/0	0/0	NC_027899.1	81192971	T	A	
30	0/1	1/1	1/1	1/1	1/1	1/1	1/1	1/1	0/0	0/0	1/1	NC_027906.1	5632534	A	G	
29	0/1	1/1	1/1	1/1	1/1	1/1	1/1	1/1	0/0	0/1	0/1	NC_027906.1	37872424	T	C	
29	0/0	0/1	1/1	1/1	0/1	1/1	1/1	0/1	0/0	0/0	0/0	NC_027911.1	45306328	G	A	
29	0/0	0/1	1/1	1/1	0/1	1/1	1/1	0/1	0/0	0/0	0/0	NC_027911.1	45315199	G	A	
29	0/0	0/1	1/1	1/1	0/1	1/1	1/1	0/1	0/0	0/0	0/0	NC_027911.1	45320549	C	T	
28	0/0	0/1	1/1	1/1	1/1	1/1	0/1	1/1	0/0	0/0	0/1	NC_027893.1	137864085	A	G	
27	0/0	0/1	1/1	0/1	0/1	0/1	1/1	1/1	0/0	0/0	0/0	NC_027894.1	104743800	T	C	
27	0/1	1/1	1/1	1/1	1/1	1/1	1/1	1/1	1/1	0/1	0/0	NC_027901.1	913673	G	C	

IL20RA or MAP3K5

Score	Donor	Response group							Non-response group		Chromosome	Nucleotide	Haplotypes		
	cy0747	cy0742	cy0743	cy0746	cy0870	cy0872	cy0874	cy0739	cy0736	cy0878			**0**	**1**	**2**
32	0/1	1/1	1/1	1/1	1/1	1/1	1/1	1/1	0/1	0/0	NC_027896.1	130270765	A	G	
32	0/1	1/1	1/1	1/1	1/1	1/1	1/1	1/1	0/1	0/0	NC_027896.1	130725262	C	G	
30	0/1	1/1	1/1	1/2	1/1	1/1	1/1	1/1	1/2	1/2	NC_027896.1	131870968		T	G
30	0/0	1/1	1/1	1/1	1/1	1/1	1/1	0/1	0/0	0/1	NC_027906.1	107528843	C	T	
30	1/2	1/1	1/1	1/1	1/1	1/1	1/1	1/1	1/1	1/2	NC_027911.1	35236508		C	A
29	0/1	1/1	1/1	1/1	1/1	1/1	1/1	1/1	0/1	0/1	NC_027893.1	33346171	C	T	
29	0/1	1/1	1/1	1/1	1/1	1/1	1/1	1/1	0/1	0/1	NC_027893.1	33346232	C	T	
29	0/1	1/1	1/1	1/1	1/1	1/1	1/1	1/1	0/1	0/1	NC_027893.1	79010358	C	A	
29	0/1	1/1	1/1	1/1	1/1	1/1	1/1	1/1	0/1	0/1	NC_027893.1	79027949	T	C	
29	0/1	1/1	1/1	1/1	1/1	1/1	1/1	1/1	0/1	0/1	NC_027893.1	142846420	A	G	

Chromosome and nucelotide locations are relative to Mmul-8.0.1 rhesus macaque reference genome
